# Plasma P‐tau217, GFAP, and NfL as biomarkers for Alzheimer's disease: role in disease stratification, pathological progression, and cognitive decline

**DOI:** 10.1002/alz.70987

**Published:** 2025-12-10

**Authors:** Feng‐Feng Pan, Lin Huang, Ying Wang, Qi Huang, Yi‐Hui Guan, Yue‐hua Li, Fang Xie, Qi‐Hao Guo

**Affiliations:** ^1^ Department of Gerontology Shanghai Sixth People's Hospital Affiliated to Shanghai Jiao Tong University School of Medicine Shanghai China; ^2^ Department of Nuclear Medicine & PET Center, Huashan Hospital Fudan University Shanghai China; ^3^ Department of Radiology Shanghai Sixth People's Hospital Affiliated to Shanghai Jiao Tong University School of Medicine Shanghai China

**Keywords:** Alzheimer's disease, amyloid PET, blood biomarkers, cognitive decline, hippocampal, tau PET

## Abstract

**INTRODUCTION:**

Blood‐based phosphorylated tau 217 (p‐tau217), glial fibrillary acidic protein (GFAP), and neurofilament light chain (NfL) show promise for Alzheimer's disease (AD), while their links to brain amyloid beta (Aβ)/tau, hippocampal atrophy, and cognitive decline need further investigation.

**METHODS:**

A cohort of 1275 participants, representing various cognitive stages, was recruited to examine the links between plasma biomarkers and brain Aβ/tau stages, tau progression, hippocampal atrophy, and cognitive decline.

**RESULTS:**

Plasma p‐tau217 effectively distinguished A−T−/A−T+ individuals and A+T+_Braak III–VI_ patients, though it identified early A+T−/A+T+_Braak I‐II_ stages only in Aβ+ subjects. Plasma GFAP levels plateau beyond a certain tau threshold, while Aβ‐induced tau progression occurred only in those with high GFAP. Plasma NfL showed a weak link to brain Aβ and tau pathology, hippocampal atrophy, and typical AD cognitive decline.

**DISCUSSION:**

Plasma p‐tau217 aids in disease stratification, and GFAP promotes tau progression, while NfL is inadequate as a neuronal injury biomarker for AD.

**Highlights:**

- Plasma p‐tau217 is strongly linked to brain Aβ/tau burdens and effectively differentiates between various Aβ/tau stages.
- Elevated plasma levels of GFAP consistently contributed to the Aβ‐induced tau progression across various Braak stages.
- Plasma NfL exhibits limited associations with Aβ/tau pathology, AD‐specific hippocampal atrophy, and cognitive decline.

## 1 BACKGROUND

In Alzheimer's disease (AD), key pathological changes involve the extracellular deposition of amyloid beta (Aβ), which forms senile plaques, and the intracellular accumulation of hyperphosphorylated tau proteins, which forms neurofibrillary tangles (NFTs).^1^ Aβ/tau positron emission tomography (PET) scans can detect these pathologies and accurately stage AD by evaluating Aβ levels and tau distribution in the brain.^2^ In 2024, the Alzheimer's Association (AA) Workgroup introduced revised diagnostic and staging criteria for AD, which included an operational framework for biological staging based on the spatial distribution and quantification of Aβ/tau PET scans.^3^ However, the extensive cost and limited accessibility of Aβ/tau PET imaging pose significant barriers to its widespread clinical implementation. Recent advancements in blood‐based biomarkers for AD offer promising potential to enhance the biological definition of the disease.

At present, the most promising blood‐based biomarkers for AD with clinical applicability include the tau phosphorylated at threonine 217 (p‐tau217), glial fibrillary acidic protein (GFAP), and neurofilament light chain (NfL), which provide multidimensional information in AD.^4^, ^5^ Studies examining the relationship between blood‐based p‐tau217 and both Aβ‐PET and tau‐PET imaging have demonstrated that p‐tau217 levels correspond to both Aβ and tau abnormalities, thereby broadly reflecting AD neuropathological changes (ADNPCs).^6^, ^7^, ^8^, ^9^, ^10^, ^11^ Nevertheless, these studies have predominantly focused on evaluating the strength of the association between p‐tau217 and either Aβ‐PET or tau‐PET in isolation. They have not extensively explored the evolution of blood‐based p‐tau217 in the context of the Aβ/tau stages in AD, as well as in populations exhibiting atypical Aβ/tau imaging patterns. Further investigation is required to ascertain whether alterations in plasma p‐tau217 levels can reliably reflect AD stages and differentiate between low‐ and high‐stage tau pathology. Elevated plasma levels of GFAP indicate astrocyte activation, which is closely associated with cerebral Aβ deposition and concurrent tau pathology.^12^ A prior study involving cognitively unimpaired individuals revealed that Aβ burden‐induced tau accumulation in the Braak I–II region was observed exclusively in subjects with increased plasma GFAP levels.^13^ Nevertheless, the evolution of plasma GFAP in relation to brain Aβ/tau burden, as well as the exact interaction between astrocyte activation and Aβ deposition in the advanced pathological stages of AD, warrants further investigation. Elevated plasma NfL levels have been frequently associated with AD, with higher concentrations correlating with reduced cognitive performance and accelerated disease progression.^14^, ^15^, ^16^ However, due to the non‐specific character of NfL as a biomarker for AD‐related neuronal injury and the common presence of comorbidities alongside AD progression,^17^, ^18^ it remains unclear whether these associations directly contribute to AD or are a consequence of comorbid conditions. Further investigation into the relationship between plasma NfL levels and Aβ/tau in advanced stages of AD, as well as the association between plasma NfL and typical AD‐related neuronal injury and cognitive phenotypes, may yield valuable insights into the role of plasma NfL in the context of AD.

To provide more compelling evidence for the clinical applicability of blood‐based biomarkers in AD, we primarily aimed to investigate the evolutionary patterns of these plasma biomarkers throughout the course of AD, assess the diagnostic accuracy of plasma p‐tau217 in distinguishing between various Aβ/tau stages, substantiate the role of plasma GFAP in the Aβ‐induced propagation of tau pathology across Braak stages, and examine the involvement of plasma NfL in AD‐associated hippocampal atrophy and cognitive deterioration.

**RESEARCH IN CONTEXT**

1. **Systematic review**: The authors utilized PubMed to perform their literature review. Although plasma p‐tau217, GFAP, and NfL exhibit considerable potential in the context of AD, there is a paucity of studies that concurrently examine their associations with brain Aβ/tau burdens and stages, hippocampal atrophy, and cognitive decline.
2. **Interpretation**: In this cross‐sectional study, we observed that plasma p‐tau217, GFAP, and NfL exhibited distinct characteristics in response to Aβ/tau pathology. Notably, plasma p‐tau217 demonstrated a strong capacity for differentiating between Aβ/tau stages; abnormal plasma GFAP appeared to facilitate Aβ‐induced tau progression, whereas plasma NfL wa more closely associated with non‐AD‐specific neuronal injury and cognitive decline.
3. **Future directions**: Our findings highlight the role of these plasma biomarkers in the progression of AD. To precisely determine their clinical utility in the classification and prognostic assessment of AD, further longitudinal studies are necessary.

## 2 METHODS

### 2.1 Study participants

Participants for our study were sourced from the Chinese Preclinical Alzheimer's Disease Study (C‐PAS) research cohort.^19^ A total of 1275 participants underwent both 3.0T brain magnetic resonance imaging (MRI) and ^18^F‐florbetapir (AV45) PET scans, along with concurrent blood sampling for the measurement of plasma p‐tau217, GFAP, and NfL, were included in the present analysis. A subset of 584 participants underwent MK6240‐PET scans. Written informed consent was obtained from all participants or their caregivers. The ethics committee of Shanghai Sixth People's Hospital, affiliated with Shanghai Jiao Tong University School of Medicine, reviewed and approved this study.

### 2.2 Cognitive assessments and clinical diagnoses

Global cognitive performance and functional status were assessed via the Montreal Cognitive Assessment‐Basic (MoCA‐B) and the Activities of Daily Living (ADL), respectively.^20^, ^21^ A comprehensive battery of standardized neuropsychological tests was administered to derive composite scores across five cognitive domains, as previously documented.^22^ In brief, memory is calculated as the average of the *z*‐scores for Auditory Verbal Learning Test delayed recall and Brief Visuospatial Memory Test‐Revised delayed recall. Language is calculated as the average of the *z*‐scores for Boston Naming Test and Animal Verbal Fluency Test. Attention is calculated as the average of the *z*‐scores for Symbol Digit Modalities Test and Digit Span Test. Visuospatial is calculated as the average of the *z*‐scores for silhouette test and judgment of line orientation. Execution is calculated as the average of the *z*‐scores for Shape Trail Test Parts A and B. The diagnosis of mild cognitive impairment (MCI) was established based on the actuarial neuropsychological criteria proposed by Jak and Bondi.^23^ The diagnosis of probable AD dementia was determined according to the criteria set forth by the National Institute on Aging‐Alzheimer's Association (NIA‐AA) in 2011.^24^ Participants who performed within the normal range and did not meet the criteria for MCI or dementia were classified as cognitively normal (CN).

### 2.3 MRI image and hippocampal volume

Brain MRI images were obtained utilizing a 3.0T MRI scanner (SIEMENS MAGNETOM Prisma 3.0T, Siemens, Erlangen, Germany) within the Department of Radiology at Shanghai Sixth People's Hospital. The volumes of the left and right hippocampus (HV‐L, HV‐R) were determined by dividing the total hippocampal volume by the estimated total intracranial volume and subsequently multiplying by 1000. For details regarding the acquisition and preprocessing of the MRI images, see Supporting Information.

### 2.4 Amyloid/tau PET imaging and AT classification

Both AV45‐PET and MK6240‐PET scans were conducted utilizing a PET/computed tomography (CT) system (Biograph mCT Flow, Siemens, Erlangen, Germany) at the Department of Nuclear Medicine & PET Center, Huashan Hospital, Fudan University. Subjects were classified as Aβ positive (A+) and negative (A−) through visual interpretation of AV45‐PET scans, following established visual rating guidelines as previously documented.^25^ The visual rating of MK6240‐PET images adhered to the guidelines for tau PET interpretation.^26^ Based on the Braak staging system,^27^ subjects were categorized into tau negative (T−) and tau positive (T+) stages, which include Braak I–II (entorhinal cortex and hippocampus), Braak III–IV (limbic and temporal neocortex), and Braak V–VI (neocortical association areas). Consequently, Aβ/tau‐PET images were classified into A−T−, A−T+, A+T−, A+T+_Braak I–II_, A+T+_Braak III–IV_, and A+T+_Braak V–VI_. Brain Aβ burden was quantified using standard uptake value ratios (SUVRs) for the global cortical region, determined through a weighted average of the region of interest (ROI) relative to the cerebellar crus. In the context of MK6240 PET imaging, SUVR was computed using the inferior cerebellar gray matter as the reference region. Both global MK6240‐SUVR and three composite regions corresponding to Braak I–II, Braak III–IV, and Braak V–VI stages were calculated independently. Comprehensive details regarding image acquisition, data preprocessing, visual interpretation, and SUVR calculation for both AV45‐PET and MK6240‐PET are provided in the Methods section of the Supporting Information.

### 2.5 Measurements of plasma biomarkers

Plasma samples were obtained from ethylenediaminetetraacetic acid (EDTA)‐treated blood and subsequently subjected to centrifugation, aliquoting, and storage at −80°C. The concentrations of plasma biomarkers, including p‐tau217, GFAP, and NfL, were quantified using the Light‐initiated Chemiluminescent Assay (LiCA) on the Chemclin LiCA 800 automated immunoassay analyzer (Chemclin Diagnostics, Beijing, China). Further description of assays is provided in the Methods in the Supporting Information.

### 2.6 Statistical analyses

Categorical variables were represented as frequencies (percentages) and analyzed utilizing the chi‐squared test. Continuous variables were presented as means  ±  standard deviation (SD), with multigroup comparisons conducted using analysis of variance (ANOVA) followed by post hoc Bonferroni correction. Segmental linear regression analysis was further conducted to compare the variation characteristics of different plasma biomarkers in relation to changes in brain Aβ and tau burden. Prior to analysis, plasma biomarkers were standardized using *z*‐scores. The area under the receiver operating characteristic (ROC) curve (AUROC) was employed to assess the efficacy of plasma biomarkers in differentiating various AT statuses. Cutpoints were determined according to the Youden index of ROC curves. A general linear model (GLM) adjusted for age and gender was utilized to evaluate the associations between Aβ and tau burden in different statuses of astrocyte activation. Linear regression analyses adjusted for age, sex, and years of education were conducted to examine the predictive effects of plasma p‐tau217 and NfL on hippocampal volume and cognitive performances. Plasma p‐tau217 and NfL were *z*‐scored before entering the analysis. Statistical significance was determined using a two‐sided *p* value threshold of <0.05. Data analyses were executed using IBM SPSS Statistics version 26 and GraphPad Prism version 9.0 (GraphPad Software, Inc.).

## 3 RESULTS

### 3.1 Participant characteristics

In this study, comprising 1275 participants, 462 individuals were classified as CN, 539 as having MCI, and 274 diagnosed with probable AD dementia. As indicated in Table 1, there was a significant rise in plasma levels of GFAP, p‐tau217, NfL, apolipoprotein E (*APOE*) ε4 prevalence, AV45‐PET positivity, and global AV45‐SUVR from the CN group to the MCI group and then to the dementia group, while hippocampal volumes significantly decreased. Among participants who underwent MK6240‐PET scanning, the prevalence of individuals at Braak V–VI stage showed a significant increase from the CN group to the MCI group and then to the dementia group. However, no significant difference was observed in the prevalence of individuals at Braak I–II and III–IV stages across the different cognitive stages. MK6240‐SUVR in Braak I–II regions showed a significant increase across cognitive stages, whereas significant increases in the global cortex and Braak III–IV and V–VI regions were observed exclusively in individuals with dementia. The global and regional MK6240‐SUVRs for subgroups categorized by the final Braak stages are illustrated in Figure S1.

**TABLE 1:** Demographics and key characteristics of participants.

Index	ALL (*n* = 1275)	CN (*n* = 462)	MCI (*n* = 539)	Dementia (*n* = 274)
Gender (male, *n* [%])	492 (38.6%)	169 (36.6%)	215 (39.9%)	108 (39.4%)
Age (years)	66.75 ± 7.93	64.30 ± 7.94	67.61 ± 7.16^a^	69.18 ± 8.28^b^, ^c^
Education (years)	11.33 ± 3.77	12.63 ± 3.23	11.18 ± 3.54^a^	9.39 ± 4.16^b^, ^c^
*APOE* ε4+ (*n* [%])	356 (27.9%)	92 (19.9%)	142 (26.3%)^a^	122 (44.5%)^b^, ^c^
MoCA‐B scores	21.28 ± 6.03	25.75 ± 2.52	21.56 ± 4.24^a^	12.80 ± 4.43^b^, ^c^
ADL scores	21.48 ± 4.78	20.22 ± 1.83	20.89 ± 3.66	24.78 ± 7.76^b^, ^c^
Hippocampal volume (L, mm^3^)	3079 ± 453	3256 ± 334	3133 ± 391^a^	2585 ± 458^b^, ^c^
Hippocampal volume (R, mm^3^)	3217 ± 482	3395 ± 360	3279 ± 408^a^	2704 ± 516^b^, ^c^
Plasma p‐tau217 (pg/mL)	0.53 ± 0.44	0.35 ± 0.17	0.45 ± 0.31^a^	1.01 ± 0.62^b^, ^c^
Plasma GFAP (pg/mL)	148.60 ± 79.68	119.76 ± 57.07	140.22 ± 66.69^a^	214.68 ± 97.70^b^, ^c^
Plasma NfL (pg/mL)	30.19 ± 14.19	25.84 ± 10.65	29.35 ± 12.02^a^	39.03 ± 18.63^b^, ^c^
AV45‐PET+ (*n* [%])	365 (28.6%)	39 (8.4%)	128 (23.7%)^a^	196 (71.5%)^b^, ^c^
AV45‐SUVR _(Global)_	1.29 ± 0.17	1.24 ± 0.11	1.27 ± 0.16^a^	1.40 ± 0.23^b^, ^c^
MK6240‐PET (*n*)	584	207	233	144
MK6240‐PET+ _(Braak I–II)_ (*n* [%])	47 (8.0%)	15 (7.2%)	23 (9.9%)	9 (6.3%)
MK6240‐PET+ _(Braak III–IV)_ (*n* [%])	36 (6.2%)	7 (3.4%)	20 (8.6%)	9 (6.3%)
MK6240‐PET+ _(Braak V–VI)_ (n [%])	121 (20.7%)	0 (0%)	32 (13.7%)^a^	89 (61.8%)^b^, ^c^
MK6240‐SUVR _(Global)_	1.12 ± 0.45	0.94 ± 0.07	0.99 ± 0.21	1.52 ± 0.69^b^, ^c^
MK6240‐SUVR _(Braak I–II)_	1.05 ± 0.38	0.85 ± 0.12	1.00 ± 0.88^a^	1.37 ± 0.45^b^, ^c^
MK6240‐SUVR _(Braak III–IV)_	1.12 ± 0.47	0.91 ± 0.08	1.00 ± 0.26	1.56 ± 0.68^b^, ^c^
MK6240‐SUVR _(Braak V–VI)_	1.08 ± 0.44	0.93 ± 0.07	0.96 ± 0.19	1.46 ± 0.69^b^, ^c^

*Note*: Data are shown as mean ±  SD or *n* (%). Group comparisons were performed using ANOVA or chi‐squared tests based on the data type. Bonferroni correction was applied to all multiple comparisons.

Abbreviations: ADL, Activities of Daily Living; APOE, apolipoprotein E; AV45, ^18^F‐florbetapir; Aβ, amyloid beta; CN, cognitively normal; GFAP, glial fibrillary acidic protein; MCI, mild cognitive impairment; MoCA‐B, Montreal Cognitive Assessment‐Basic; NfL, neurofilament light chain; PET, positron emission tomography; SUVR, standardized uptake value ratio.

^a^ Different from CN and MCI.

^b^ Different from CN and dementia.

^c^ Different from MCI and dementia.

### 3.2 Non‐linear evolution of plasma biomarkers in response to increasing amyloid/tau burdens

**FIGURE 1:**
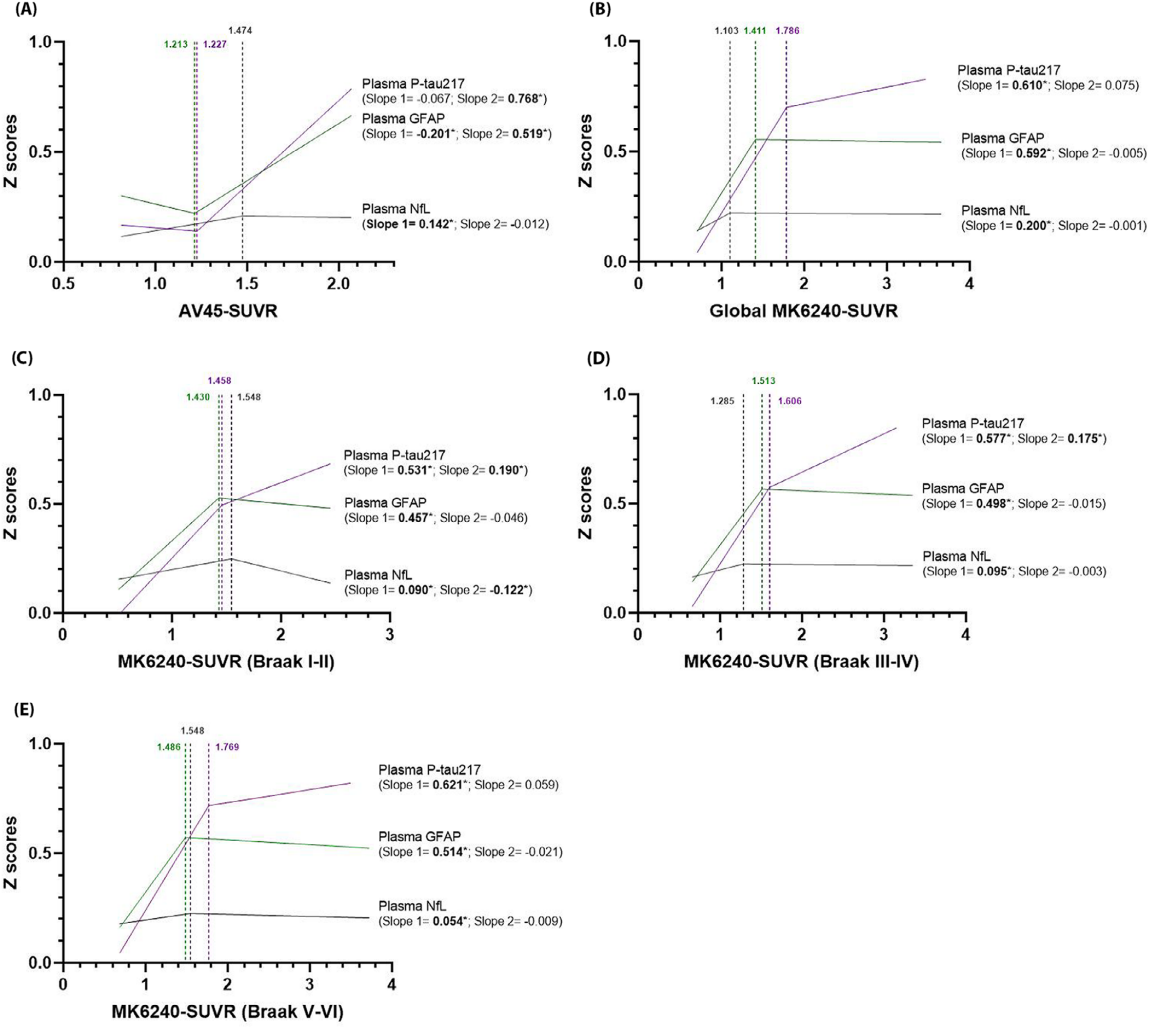
Non‐linear evolution of plasma biomarkers in response to increasing Aβ and tau burdens. (A) Aβ burdens are measured by global AV45‐SUVR (*n* = 1275). (B–E) Tau burdens are assessed using the global MK6240‐SUVR, as well as MK6240‐SUVRs across different Braak stage regions of interest (*n* = 584). Plasma biomarkers were *z*‐scored before analysis. The lines depicted represent trends derived from segmental linear regression analysis. Vertical dashed lines and their corresponding scores were obtained from the optimal fit of the regression models. Slopes 1 and 2 indicate the regression slopes to the left and right of the vertical dashed lines, respectively. Asterisks indicate statistical significance. Aβ, amyloid beta; SUVR, standardized uptake value ratio.

Segmental linear regression analysis was employed to investigate the progression of plasma biomarkers in relation to increasing amyloid/tau burdens. Breakpoints indicating significant changes were identified using the least‐squares method, which minimized the residual sum of squares. As shown in Figure 1A, plasma p‐tau217 did not exhibit significant correlation with AV45‐SUVR levels below the breakpoint but increased gradually once the breakpoint was exceeded (Slope 2 = 0.768). Plasma GFAP initially demonstrated a transient decrease prior to the breakpoint, followed by a consistent increase (Slope 1 = −0.201, Slope 2 = 0.519). In contrast, plasma NfL levels showed a significant yet weak increase before the breakpoint (Slope 1 = 0.142) and did not exhibit a progressive increase with further elevations in AV45‐SUVR. Figure 1B–E illustrates the trends in plasma biomarkers as MK6240‐SUVR levels rise. Plasma p‐tau217 levels exhibited a progressive rise with increasing global MK6240‐SUVR (Slope 1 = 0.610), until a high SUVR threshold was reached, beyond which the rising trend ceased to be significant. Plasma GFAP levels showed a progressive rise with increasing global MK6240‐SUVR (Slope 1 = 0.592), followed by a plateau beyond a breakpoint, which was identified at a lower SUVR value compared to that of p‐tau217. In the Braak regions, plasma GFAP displayed similar trends, while plasma p‐tau217 demonstrated distinct patterns in the Braak I–II and Braak III–IV regions. Specifically, plasma p‐tau217 demonstrated a progressive increase with MK6240‐SUVR before reaching the breakpoints (Slope 1 = 0.531 and 0.577, respectively) and continued to show a relatively modest yet statistically significant upward trend after the breakpoints (Slope 2 = 0.190 and 0.175, respectively). Regarding plasma NfL, aside from a slight increase observed before the breakpoints, no elevation in NfL levels was associated with increasing MK6240‐SUVR. Figure S2 shows a scatterplot of plasma biomarkers with AV45‐SUVR and MK6240‐SUVR.

### 3.3 Plasma biomarkers and PET‐defined A/T statuses

**FIGURE 2:**
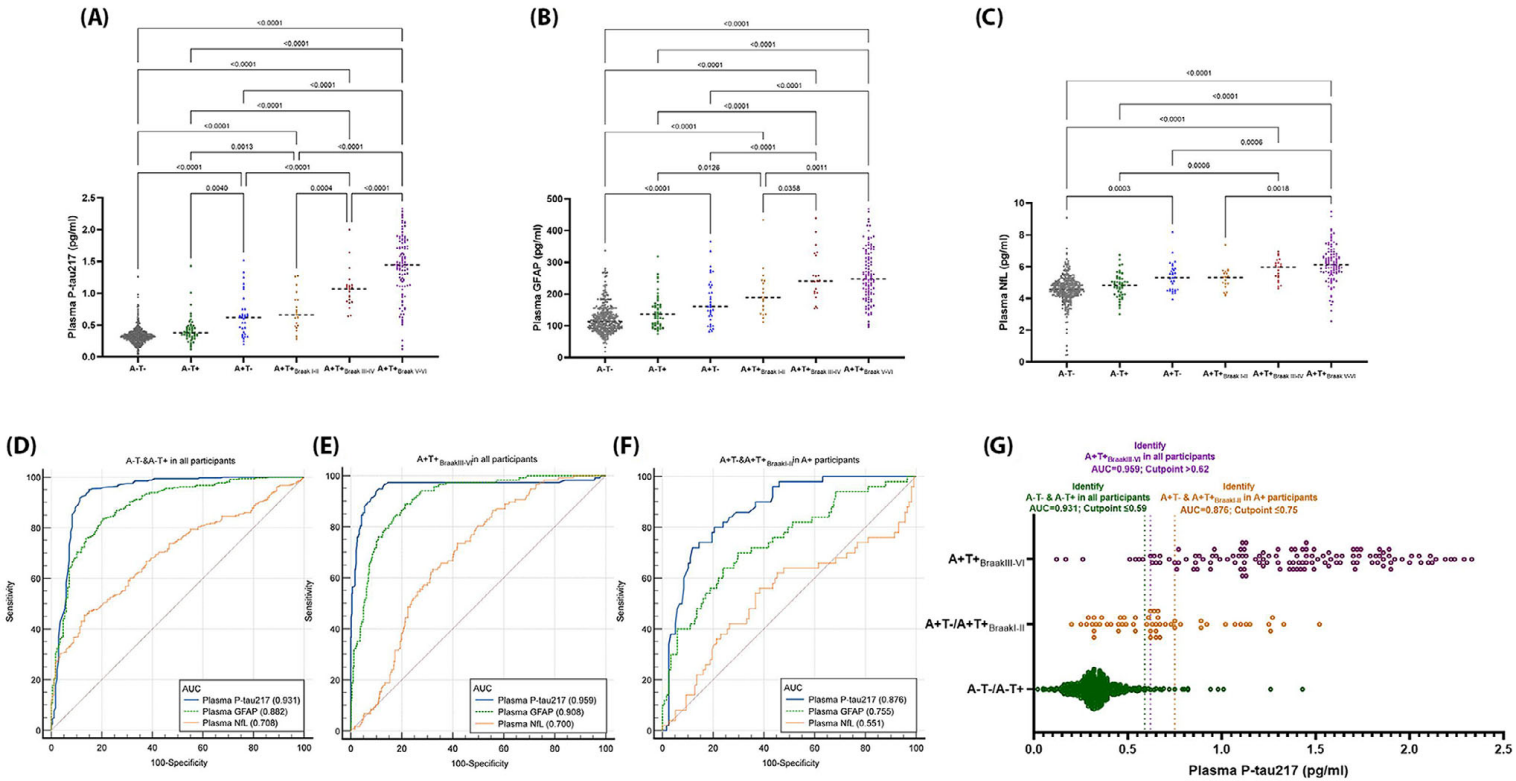
Associations between plasma biomarkers and PET‐defined Aβ/tau (AT) statuses. (A–C) Differences in plasma biomarkers across different AT statuses and AD progression stages (*n* = 584). (D–F) ROC analyses for the plasma biomarkers in detecting A−T− and A−T+ and A+T+_Braak III–VI_ in all participants (*n* = 584), and discriminating A+T− and A+T+_Braak I–II_ with A+T+_Braak III–VI_ in A+ participants (*n* = 190). (G) Ability of plasma p‐tau217 in discriminating various stages of AT progression. AT statuses are defined by AV45‐PET and MK6240‐PET on the basis of visual reading. Group comparisons are performed using the ANOVA with Bonferroni correction. AD, Alzheimer's disease; ANOVA, analysis of variance; AUC, area under the curve; GFAP, glial fibrillary acidic protein; NfL, neurofilament light chain; PET, positron emission tomography; p‐tau217, tau phosphorylated at threonine 217; ROC, receiver operating characteristic.

The differences in plasma biomarkers across the different A/T statuses defined by Aβ/tau‐PET are shown in Figure 2A–C. Plasma p‐tau217 and GFAP levels were elevated in all A+ groups relative to the A−T− and A−T+ groups, with the exception of GFAP levels, which showed no significant difference between the A+T− and A−T+ groups. Moreover, plasma p‐tau217 and GFAP concentrations were significantly higher in the A+T+_Braak III–IV_ and A+T+_Braak V–VI_ groups compared to the A+T− and A+T+_Braak I–II_ groups. No significant differences were noted between the A−T− and A−T+ groups or between the A+T− and A+T+_Braak I–II_ groups. Additionally, plasma p‐tau217 levels were elevated in the A+T+_Braak V–VI_ group compared to the A+T+_Braak III–IV_ group. Compared to the A−T− group, plasma NfL showed a slight increase in the A+T− group, and the elevation in NfL levels in the A+T+_Braak III–IV_ and A+T+_Braak V–VI_ groups was not as pronounced as the increases observed in p‐tau217 and GFAP.

Given that the primary beneficiaries of current anti‐Aβ AD‐modifying therapies are likely to be the A+T− and A+T+_Braak I–II_ groups, we assessed the efficacy of plasma biomarkers in distinguishing these populations. ROC analyses indicated that plasma p‐tau217 exhibited the highest discriminative capacity among the various AT groups (Figure 2D–F and Table S1). Within the overall population, plasma p‐tau217 demonstrated a robust ability to exclude both the A−T− and A−T+ groups (area under the curve, AUC = 0.931), while also effectively identifying the A+T+_Braak III–IV_ and A+T+_Braak V–VI_ groups (AUC = 0.959). However, because the plasma p‐tau217 levels in individuals with A+T− and A+T+_Braak I–II_ largely overlapped with those in either the A−T− and A−T+ groups or the A+T+_Braak III–IV_ and A+T+_Braak V–VI_ groups (Figure 2G), it could not effectively distinguish individuals with A+T− and A+T+_Braak I–II_ in all the participants. Nevertheless, plasma P‐tau217 retained a high ability to differentiate the A+T− and A+T+_Braak I–II_ groups within the Aβ‐PET‐positive population (AUC = 0.876).

### 3.4 Astrocyte activation on amyloid‐induced tau pathology

**FIGURE 3:**
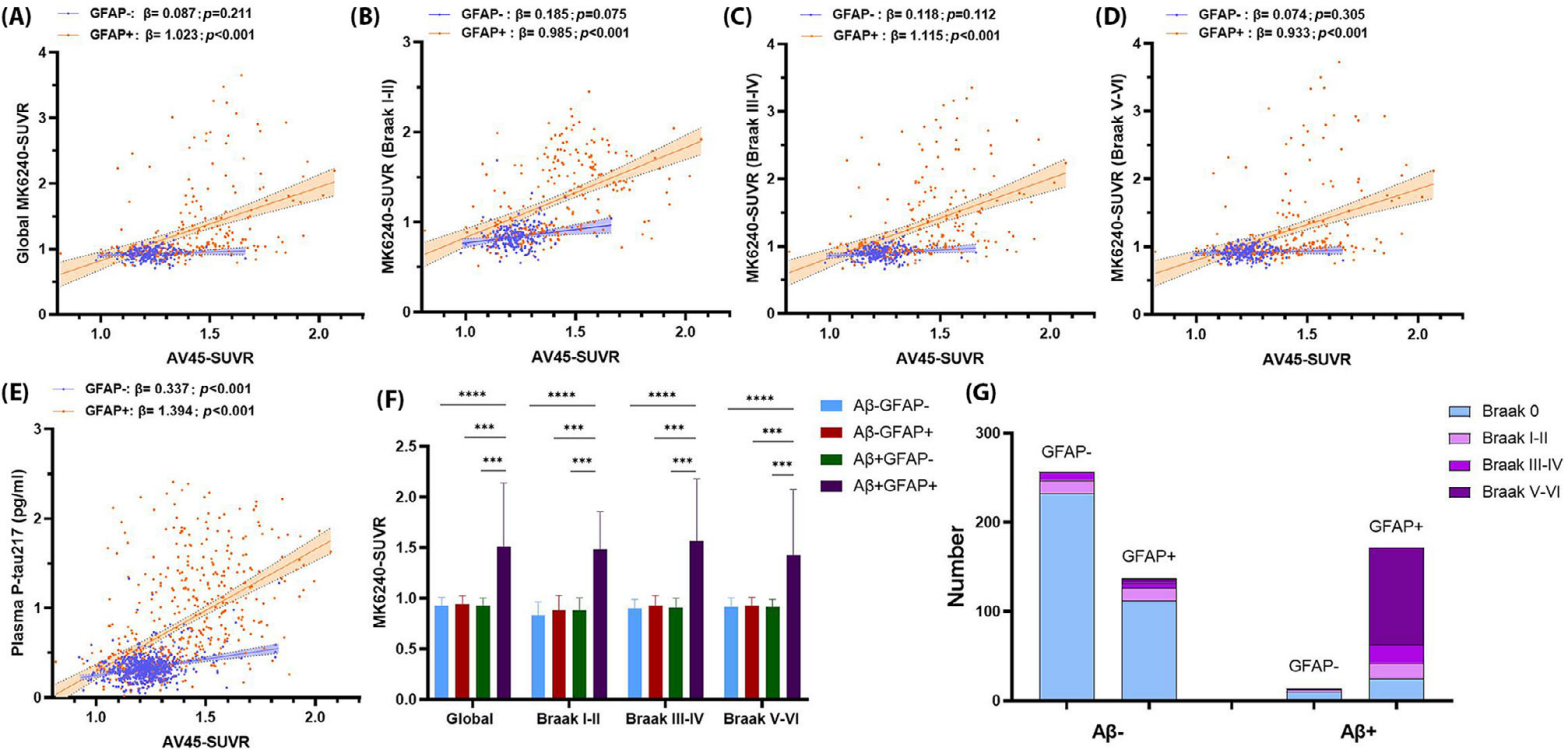
Effects of astrocyte activation on amyloid‐induced tau pathological progression. (A–E) Effects of AV45‐SUVR on MK6240‐SUVR in global cortex and specific Braak regions (*n* = 584), as well as plasma p‐tau217 levels (*n* = 1275) in GFAP− and GFAP+ participants, adjusted for age and sex. (F) Differences of MK6240‐SUVR between different Aβ deposition and astrocyte activation statuses (***p < 0.001) (*n* = 584). (G) Astrocyte activation associated distribution of tau pathological progression (Braak stages 0–VI) in different Aβ deposition statuses (*n* = 584). Astrocyte activation statuses (GFAP+ and GFAP−) are determined by the 95th‐percentile level of plasma GFAP in cognitively normal young controls who are Aβ‐negative within the overall C‐PAS cohort. Aβ deposition statuses (Aβ+ and Aβ−) are determined by AV45‐PET based on visual reading. Aβ, amyloid beta; C‐PAS, Chinese Preclinical Alzheimer's Disease Study; GFAP, glial fibrillary acidic protein; p‐tau217, tau phosphorylated at threonine 217; SUVR, standardized uptake value ratio.

Plasma GFAP elevation serves as an indicator of astrocyte activation. Within the comprehensive C‐PAS cohort, astrocyte activation is defined by the 95th‐percentile level of plasma GFAP in CN young controls who are Aβ− (annotated as GFAP+). After adjusting for age and gender, there was a significant increase in global MK6240‐SUVR corresponding to the rise in AV45‐SUVR within the GFAP+ population (Figure 3A; *β* = 1.023, *p* < 0.001). Conversely, in the GFAP− population, the increase in AV45‐SUVR did not significantly impact global MK6240‐SUVR (*β* = 0.087, *p* = 0.211). A similar pattern was found in the distinct Braak regions for both GFAP+ and GFAP− populations (Figure 3B–D). Additionally, the similar impact of AV45‐SUVR on plasma p‐tau217 levels was also observed in the GFAP− and GFAP+ populations (Figure 3E). Figure 3F illustrates the variations in MK6240‐SUVR across the Aβ and GFAP subgroups in the global cortex and distinct Braak regions, revealing a significant increase in MK6240‐SUVR exclusively in the cohort positive for both Aβ and GFAP. Furthermore, Figure 3G indicates that tau pathology beyond Braak stage III was infrequent in populations negative for both Aβ and GFAP or when only one marker was positive. In contrast, this was more prevalent in the population with concurrent Aβ+ and GFAP+.

### 3.5 Associations of plasma p‐tau217 and NfL with hippocampal volume and cognitive function

**FIGURE 4:**
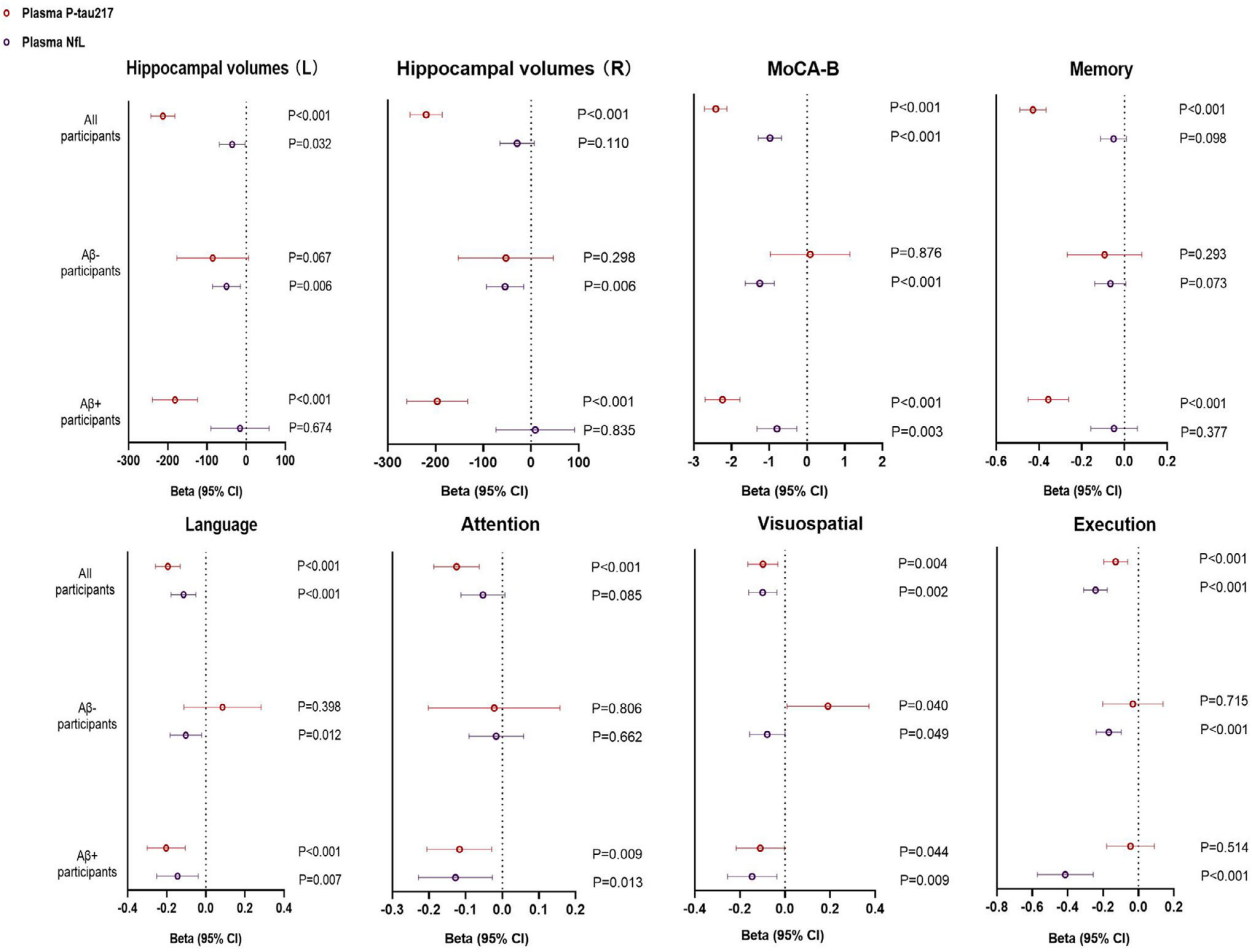
Effects of plasma P‐tau217 and NfL on predicting hippocampal volume, global cognitive decline, and performances across various cognitive domains. Beta coefficients (95% CI) and *p* values are for overall tests in linear regressions adjusted for age, sex, and years of education (*n* = 1275). Plasma p‐tau217 and NfL are *z*‐scored. Aβ‐positive is defined by AV45‐PET on the basis of visual reading. Memory is calculated as the average of the *z*‐scores for Auditory Verbal Learning Test delayed recall and Brief Visuospatial Memory Test‐Revised delayed recall. Language is calculated as the average of the *z*‐scores for Boston Naming Test and Animal Verbal Fluency Test. Attention is calculated as the average of the *z*‐scores for Symbol Digit Modalities Test and Digit Span Test. Visuospatial is calculated as the average of the *z*‐scores for silhouette test and judgment of line orientation. Execution is calculated as the average of the *z*‐scores for Shape Trail Test Parts A and B. Aβ, amyloid beta; CI, confidence interval; NfL, neurofilament light chain; PET, positron emission tomography; p‐tau217, tau phosphorylated at threonine 217.

The associations of plasma p‐tau217 and NfL on hippocampal volume, global cognitive decline, and performances across diverse cognitive domains were assessed via linear regression analyses, with age, gender, and years of education included as covariates (Figure 4 and Table S2). *Z*‐scored plasma p‐tau217 and NfL were incorporated into the models concurrently, and no collinearity between variables was observed (VIF range, 1.066 to 1.361). Among all participants, elevated plasma p‐tau217 levels demonstrated a significant correlation with reduced hippocampal volumes, whereas the association between increased plasma NfL levels and hippocampal volume reduction was only marginally significant. When stratified by Aβ status, the significant relationship between elevated plasma p‐tau217 levels and hippocampal volume reduction was evident exclusively in the Aβ+ group, with no such association observed in the Aβ− group. Conversely, increased plasma NfL levels were associated with hippocampal volume reduction solely in the Aβ− group, with no significant association found in the Aβ+ group. In relation to cognitive performance, increased plasma p‐tau217 levels were linked to declines in overall cognitive function and each of the cognitive domains. However, within subgroups categorized by Aβ status, the association between elevated plasma p‐tau217 and cognitive decline was predominantly observed in the Aβ+ group, where it demonstrated significant correlations with declines in overall cognitive function, as well as in memory, language, and attention. Conversely, elevated plasma NfL was primarily associated with declines in overall cognitive function and language, visuospatial, and executive abilities, with these associations evident in both Aβ− and Aβ+ groups. Importantly, within the Aβ+ group, elevated plasma p‐tau217, as opposed to plasma NfL, was significantly associated with memory decline, whereas plasma NfL, rather than p‐tau217, was significantly associated with declines in executive function.

## 4 DISCUSSION

In this study, plasma p‐tau217 demonstrated a progressive increase corresponding with rising Aβ burden and exhibited a rapid‐then‐slow upward trend during the accumulation of tau pathology, while plasma GFAP tends to plateau once tau pathology surpasses a certain threshold. For distinguishing AT stages, plasma p‐tau217 may effectively exclude A−T− and A−T+ individuals and identify A+T+_Braak III–VI_ patients among all participants, while its efficacy in differentiating early stages of A+T− and A+T+_Braak I–II_ is particularly evident among the Aβ+ subjects confirmed via Aβ‐PET. Our analysis also indicated that a significant correlation between brain Aβ and tau burdens was observed exclusively in participants exhibiting abnormal plasma GFAP levels, suggesting that Aβ deposition and astrocyte activation may concurrently drive tau pathology progression. Additionally, elevated plasma p‐tau217 levels strongly predict hippocampal atrophy and global cognitive and memory decline, especially in Aβ+ individuals, highlighting its importance as an AD progression marker. In contrast, high plasma NfL levels are associated with hippocampal atrophy in Aβ− individuals and are more linked to declines in executive function rather than memory, indicating non‐AD‐related neurodegeneration and cognitive decline.

Previous research has established a strong association of plasma p‐tau217, 181, and 231 levels with Aβ/tau pathology in AD.^6^, ^8^, ^28^, ^29^, ^30^ Nevertheless, the 2024 revised AD criteria by the AA primarily categorized these biomarkers only as the indicators of Aβ stage.^3^ This is likely due to the limited evaluation of these biomarkers across various AT stages in AD. Our study indicated that while initial elevations in plasma p‐tau217 levels were found in the A+T− and A+T+_Braak I–II_ groups, a more pronounced increase in plasma p‐tau217 was observed in the A+T+_Braak III–VI_ groups. Taking together the progressively elevated levels of plasma p‐tau217 alongside increasing MK6240‐SUVR, plasma p‐tau217 may not be confined to its role as a Core 1 biomarker but also serve as a potential biomarker for advanced tau accumulation in AD. A separate addendum to Study AACI suggests that populations exhibiting no or very low tau pathology, analogous to A+T− and A+T+_Braak I–II_ stages, demonstrate a more favorable therapeutic response to anti‐Aβ treatment (https://www.fda.gov/media/179167/download). In our study, plasma p‐tau217 demonstrated high accuracy in identifying and excluding A−T−/A−T+ (AUC = 0.931) individuals and A+T+_Braak III–VI_ patients (AUC = 0.959). However, due to the proximity of the cutoff values, plasma p‐tau217 cannot effectively differentiate A+T−/A+T+_Braak I–II_ patients from the overall cohort. Nevertheless, in the cohort confirmed as Aβ+ via AV45‐PET image, plasma p‐tau217 showed high accuracy in identifying the individuals with A+T−/A+T+_Braak I–II_, achieving an AUC of 0.876. Taking together, we propose that plasma p‐tau217 be initially employed to exclude Aβ− individuals and subsequently used in conjunction with Aβ‐PET scans to accurately identify patients with early AD pathology. This strategy may be effective in precisely targeting suitable candidates for anti‐Aβ therapies. It is noteworthy that no significant elevation in plasma p‐tau217 levels was observed in the A−T+ group compared to the A−T− group. This finding is consistent with previous research^31^ and further supports the notion that plasma p‐tau217 is more indicative of AD‐related tau pathology than tau accumulation in the absence of significant Aβ deposition, commonly referred to as primary age‐related tauopathy.^32^

The overexpression of GFAP is recognized as one of the most indicative biomarkers for astrocyte reactivity.^33^ In this study, by integrating subgroups with varying Aβ and GFAP statuses, we further corroborated that significant increases in brain tau pathology burden and Braak staging were predominantly concentrated in individuals exhibiting both Aβ deposition and abnormal GFAP levels. This finding supports the hypothesis that the interaction between Aβ deposition and astrocyte reactivity plays a pivotal role in the progression of tau pathology. It is noteworthy that the significant correlation between Centiloids and MK6240‐SUVR, as influenced by abnormal plasma GFAP levels and reported by Bellaver et al.,^13^ was observed exclusively in the early Braak regions, with no such association detected in the later Braak regions. This may be attributed to their study population, which predominantly consisted of cognitively unimpaired individuals. In contrast, our study, which encompasses participants with diverse cognitive statuses, reveals that while plasma GFAP levels reach a plateau during advanced tau deposition, abnormal plasma GFAP levels persist in mediating the increase in MK6240‐SUVR as a function of Aβ burden in the later Braak regions.

Our research indicates that plasma NfL has a weak correlation with the core pathologies of AD, as it only showed a slight increase during the early stages of AV45/MK6240 SUVR elevation. To conduct a comprehensive analysis of the relationship between plasma NfL levels and neuronal injury as well as cognitive impairment in AD, we integrated plasma p‐tau217 as a predictive biomarker and concurrently evaluated the associations of both biomarkers with hippocampal atrophy and cognitive phenotypes. Our findings revealed that elevated plasma p‐tau217 levels were correlated with reduced hippocampal atrophy and exhibited a significant association with overall cognitive decline and memory impairment in the Aβ+ cohort. In contrast, plasma NfL levels in the Aβ+ group were mainly associated with non‐memory cognitive deficits, especially in executive function, and showed no significant correlation with hippocampal atrophy. However, higher plasma NfL levels were associated with hippocampal atrophy and exhibited a greater predictive value for overall cognitive impairment in the Aβ− group. Considering that plasma NfL demonstrates less variability during Aβ/tau accumulation, it is plausible that plasma NfL reflects more the comorbidities in AD rather than AD itself in relation to neuronal injury and cognitive impairment.

This study's limitations should be noted. First, due to the study's single‐center design, it is essential to validate the findings across diverse populations to ensure broader applicability. Second, the study cohort has not yet undergone adequate follow‐up, necessitating further investigation into the longitudinal trajectories of plasma biomarkers in relation to AD progression, and their levels in predicting disease progression require further confirmation. Third, although patients with severe neurological diseases other than AD were excluded, the inability to accurately ascertain potential comorbidities among participants may introduce uncertainty into the results, particularly concerning plasma NfL as a marker of neuronal damage and cognitive decline.

In conclusion, this study highlights the utility of blood‐based biomarkers, with a particular emphasis on plasma p‐tau217, in detecting Aβ/tau stages, GFAP‐mediated Aβ‐induced tau progression, and NfL‐associated non‐AD‐specific neuronal injury and cognitive decline. These findings support the implementation of plasma biomarker strategies for diagnosis, disease stratification, and prognostic prediction in Alzheimer's disease.

## CONFLICT OF INTEREST STATEMENT

The authors declare no conflict of interest. All author disclosures are available in the Supporting Information.

## CONSENT STATEMENT

This study was approved by the ethics committee of Shanghai Sixth People's Hospital and performed in accordance with the ethical standards stated in the 1964 Declaration of Helsinki and subsequent amendments.

## Supporting information



Supporting information

Supporting information
