# Supplementary material for: Plasma P‐tau217, GFAP, and NfL as biomarkers for Alzheimer's disease: role in disease stratification, pathological progression, and cognitive decline
Source: Alzheimers Dement. 2025 Dec 10;21(12):e70987. doi: 10.1002/alz.70987 (PMC12696028; doi:10.1002/alz.70987)
Supplement: Supplementary file 1 — Supporting information [file ALZ-21-e70987-s002.docx]

**Supplemental Online Content**

**eMethods.**

**eFigure 1.** Global and regional MK6240-SUVRs for subgroups categorized by the final Braak stages.

**eFigure 2.** Scatterplot of plasma biomarkers and AV45 and MK6240-SUVR in global cortex and specific Braak regions.

**eTable 1.** ROC analyses for the plasma biomarkers in discriminating various AT statuses.

**eTable 2.** Effects of plasma P-tau217 and NfL on predicting hippocampal volume, global cognitive decline, and performances across various cognitive domains.

**eMethods.**

1. **MRI image acquisition and processing**

MRI images were acquired using a 3.0 T MRI scanner (SIEMENS MAGNETOM Prisma 3.0T, Siemens, Erlangen, Germany). 3D T1-weighted MRI images were processed using the automated analysis pipeline of Freesurfer v6.0 (http://freesurfer.net/). The preprocessing steps included format conversion, motion correction, removal of non-brain tissue, affine transformation to the Talairach atlas, intensity normalization, gray-white matter segmentation, automatic topological correction, and surface deformation.

1. **PET image acquisition and processing**

PET scans using [18F]AV45 were conducted 50 minutes following an intravenous administration of approximately 370 MBq (±10%) of 18F-florbetapir, with each scan lasting 20 minutes. [18F]MK6240-PET scans were performed 90 to 110 minutes post-intravenous injection of approximately 185 MBq (±10%) of [18F]MK6240. The PET images were reconstructed utilizing a filtered back projection algorithm with corrections for decay, normalization, dead time, photon attenuation, scatter, and random coincidences. Image preprocessing was executed using Statistical Parametric Mapping12 (SPM12) software (Welcome Trust Centre for Neuroimaging, London, UK; https://www.fil.ion.ucl.ac.uk/spm). The PET images were coregistered to individual T1-weighted MRI scans, and partial volume error correction (PVC) was applied using the Muller-Gartner method(1, 2). Subsequently, the T1-weighted images were normalized to the standard Montreal Neurological Institute (MNI) space and segmented into gray matter (GM), white matter (WM), and cerebrospinal fluid (CSF). The derived normalization parameters were then applied to the corresponding PET images. The normalized PET and T1-weighted images were smoothed via a Gaussian filter with a full width at half maximum (FWHM) of 8 mm. For the analysis of volumes of interest (VOI), the PET images were subjected to spatial and intensity normalization without smoothing. In the case of the [18F]MK6240-PET data, the meninges were masked using gray matter (GM) segmentation to reduce the potential interference of meningeal spillover into adjacent cerebral regions(3).

The visual interpretation of all AV45 and MK6240 PET images was independently conducted by three nuclear medicine physicians according to the established visual rating guidelines for the interpretation of amyloid and tau PET scans(4, 5). For the [18F]MK6240 PET images, the evaluators concentrated on eight predefined brain regions in each hemisphere of the cerebral cortex, totaling 16 regions: the hippocampus, mesial temporal, inferior temporal, lateral temporal, parietal, posterior cingulate, occipital, and frontal lobes(6, 7). In this study, Braak staging was adapted to incorporate tau PET findings in the hippocampus, aligning with the methodology outlined by Schöll et al. (2016)(8). Specifically, Braak stage I-II was defined by tau deposition restricted to the entorhinal cortex and hippocampus, Braak stage III-IV was characterized by tau presence in the limbic and temporal neocortex, and Braak stage V-VI was identified by tau spread to the neocortical association areas.

The brain Aβ burden, quantified as AV45 standardized uptake value ratio (SUVR), was determined through weighted averaging across regions of interest (ROIs), including the frontal, lateral parietal, lateral temporal, medial temporal, occipital, precuneus, and posterior cingulate areas, with reference to the cerebellar crus. Global and three composite MK6240-SUVR values corresponding to Braak stages were computed using the inferior cerebellar gray matter as the reference region: Braak stages I-II (entorhinal cortex, hippocampus), III-IV (parahippocampal gyrus, fusiform gyrus, amygdala, inferior temporal gyrus, middle temporal gyrus), and V-VI (posterior cingulate cortex, caudal anterior cingulate cortex, rostral anterior cingulate cortex, precuneus, inferior parietal lobule, superior parietal lobule, insula, supramarginal gyrus, lingual gyrus, superior temporal gyrus, medial orbitofrontal cortex, rostral middle frontal gyrus, lateral orbitofrontal cortex, caudal middle frontal gyrus, superior frontal gyrus, lateral occipital cortex, precentral gyrus, postcentral gyrus, and paracentral gyrus)(9, 10).

1. **Plasma P-tau217, GFAP, and NfL Assays**

Prior to analysis, all plasma samples underwent a single freeze-thaw cycle. Based on the Light-initiated chemiluminescent assay (LiCA®), a dual-antibody sandwich technique with two-step incubations was employed for biomarker detection. Initially, the plasma sample was incubated with Chemibeads coated with detection antibodies and biotinylated capture antibodies to form a sandwich immunocomplex. Subsequently, Sensibeads conjugated with streptavidin were added to bind to the biotin on the immunocomplex. Upon excitation by a 680 nm laser, the Sensibeads generated singlet oxygen molecules, which diffused into the Chemibeads, thereby initiating a chemiluminescence reaction. Concentrations of plasma biomarkers, expressed in pg/ml, were determined by fitting to a standard curve. The biomarker measurements were conducted by laboratory technicians who were blinded to the clinical data.

Prior to sample testing, we conducted a full precision profile evaluation of the assays according to the CLSI EP05-A3 guideline. This study utilized an extensive 3×5×5 design to test two levels of quality controls and three patient plasma samples. The results demonstrated that all biomarkers exhibited excellent precision across all tested conditions. The detailed coefficients of variation (CVs) are as follows:

- The coefficients of variation (CVs) for the repeatability, within-laboratory precision, and between-laboratory precision of the LiCA^®^ p-tau 217 assay.

| Sample | Mean | Repeatability | | Within-Lab  Precision | | Between--Lab  Precision | |
| --- | --- | --- | --- | --- | --- | --- | --- |
|  | pg/mL | SD | %CV | SD | %CV | SD | %CV |
| plasma 1 | 0.43 | 0.02 | 4.21 | 0.02 | 4.47 | 0.02 | 4.86 |
| plasma 2 | 1.95 | 0.08 | 3.99 | 0.08 | 3.99 | 0.10 | 5.12 |
| plasma 3 | 4.86 | 0.15 | 3.01 | 0.15 | 3.01 | 0.15 | 3.01 |
| QC L | 1 | 0.05 | 4.87 | 0.05 | 4.87 | 0.05 | 4.87 |
| QC H | 4.98 | 0.14 | 2.77 | 0.14 | 2.87 | 0.15 | 2.96 |

- The coefficients of variation (CVs) for the repeatability, within-laboratory precision, and between-laboratory precision of the LiCA^®^ GFAP assay.

| Sample | Mean | Repeatability | | Within-Lab  Precision | | Between--Lab  Precision | |
| --- | --- | --- | --- | --- | --- | --- | --- |
|  | pg/mL | SD | %CV | SD | %CV | SD | %CV |
| plasma 1 | 91.31 | 4.22 | 4.63 | 4.38 | 4.80 | 4.47 | 4.90 |
| plasma 2 | 271.86 | 12.30 | 4.52 | 12.30 | 4.52 | 15.57 | 5.73 |
| plasma 3 | 506.92 | 14.80 | 2.92 | 14.80 | 2.92 | 14.82 | 2.92 |
| QC L | 38.91 | 2.70 | 6.93 | 2.70 | 6.93 | 2.71 | 6.96 |
| QC H | 353.91 | 10.00 | 2.83 | 10.13 | 2.86 | 10.13 | 2.86 |

- The coefficients of variation (CVs) for the repeatability, within-laboratory precision, and between-laboratory precision of the LiCA^®^ NfL assay.

| Sample | Mean | Repeatability | | Within-Lab  Precision | | Between--Lab  Precision | |
| --- | --- | --- | --- | --- | --- | --- | --- |
|  | pg/mL | SD | %CV | SD | %CV | SD | %CV |
| plasma 1 | 16.11 | 0.74 | 4.60 | 0.80 | 4.95 | 0.81 | 5.01 |
| plasma 2 | 77.91 | 2.86 | 3.68 | 2.98 | 3.82 | 3.20 | 4.10 |
| plasma 3 | 373.45 | 10.41 | 2.79 | 10.72 | 2.87 | 10.72 | 2.87 |
| QC L | 10.06 | 0.49 | 4.85 | 0.52 | 5.17 | 0.52 | 5.17 |
| QC H | 99.59 | 2.96 | 2.97 | 2.99 | 3.00 | 3.14 |  |

**eReferences**

1. Gonzalez-Escamilla G, Lange C, Teipel S, Buchert R, Grothe MJ, Alzheimer's Disease Neuroimaging I. PETPVE12: an SPM toolbox for Partial Volume Effects correction in brain PET - Application to amyloid imaging with AV45-PET. Neuroimage. 2017;147:669-77.

2. Greve DN, Salat DH, Bowen SL, Izquierdo-Garcia D, Schultz AP, Catana C, et al. Different partial volume correction methods lead to different conclusions: An (18)F-FDG-PET study of aging. Neuroimage. 2016;132:334-43.

3. Betthauser TJ, Cody KA, Zammit MD, Murali D, Converse AK, Barnhart TE, et al. In Vivo Characterization and Quantification of Neurofibrillary Tau PET Radioligand (18)F-MK-6240 in Humans from Alzheimer Disease Dementia to Young Controls. J Nucl Med. 2019;60(1):93-9.

4. Lundeen TF, Seibyl JP, Covington MF, Eshghi N, Kuo PH. Signs and Artifacts in Amyloid PET. Radiographics. 2018;38(7):2123-33.

5. Seibyl JP, DuBois JM, Racine A, Collins J, Guo Q, Wooten D, et al. A Visual Interpretation Algorithm for Assessing Brain Tauopathy with (18)F-MK-6240 PET. J Nucl Med. 2023;64(3):444-51.

6. Shuping JL, Matthews DC, Adamczuk K, Scott D, Rowe CC, Kreisl WC, et al. Development, initial validation, and application of a visual read method for [(18)F]MK-6240 tau PET. Alzheimers Dement (N Y). 2023;9(1):e12372.

7. Krishnadas N, Huang K, Schultz SA, Dore V, Bourgeat P, Goh AMY, et al. Visually Identified Tau 18F-MK6240 PET Patterns in Symptomatic Alzheimer's Disease. J Alzheimers Dis. 2022;88(4):1627-37.

8. Schöll M, Lockhart Samuel N, Schonhaut Daniel R, O’Neil James P, Janabi M, Ossenkoppele R, et al. PET Imaging of Tau Deposition in the Aging Human Brain. Neuron. 2016;89(5):971-82.

9. Cho H, Choi JY, Hwang MS, Kim YJ, Lee HM, Lee HS, et al. In vivo cortical spreading pattern of tau and amyloid in the Alzheimer disease spectrum. Ann Neurol. 2016;80(2):247-58.

10. Wang J, Huang Q, Chen X, You Z, He K, Guo Q, et al. Tau pathology is associated with synaptic density and longitudinal synaptic loss in Alzheimer’s disease. Molecular Psychiatry. 2024.


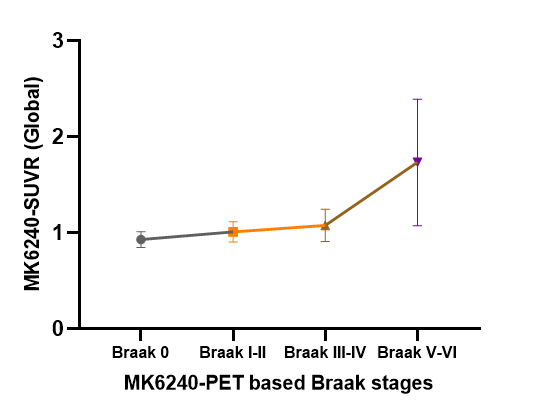

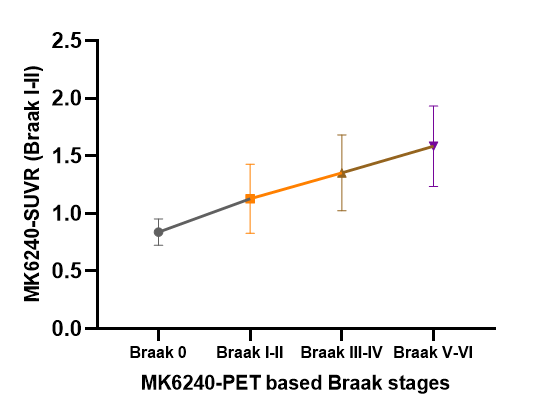

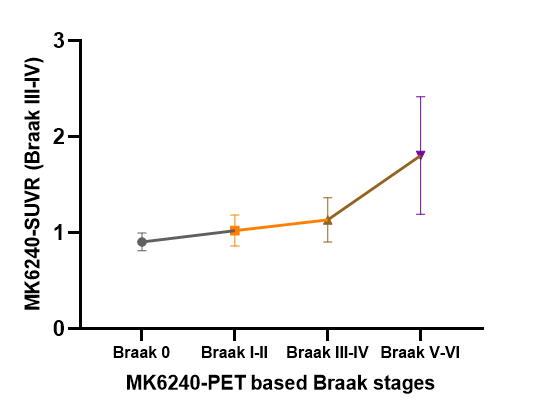

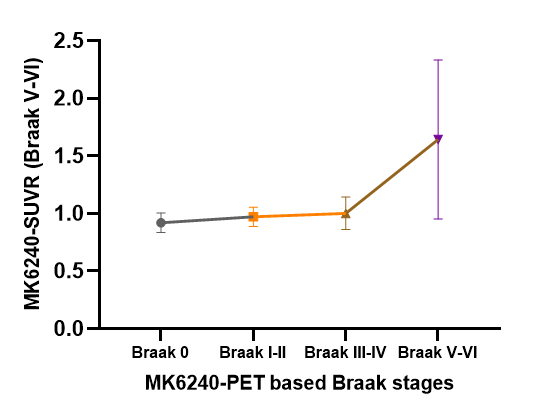


eFigure 1. Global and regional MK6240-SUVRs for subgroups categorized by the final Braak stages. Group means are represented by shapes, and error bars represent standard deviation.


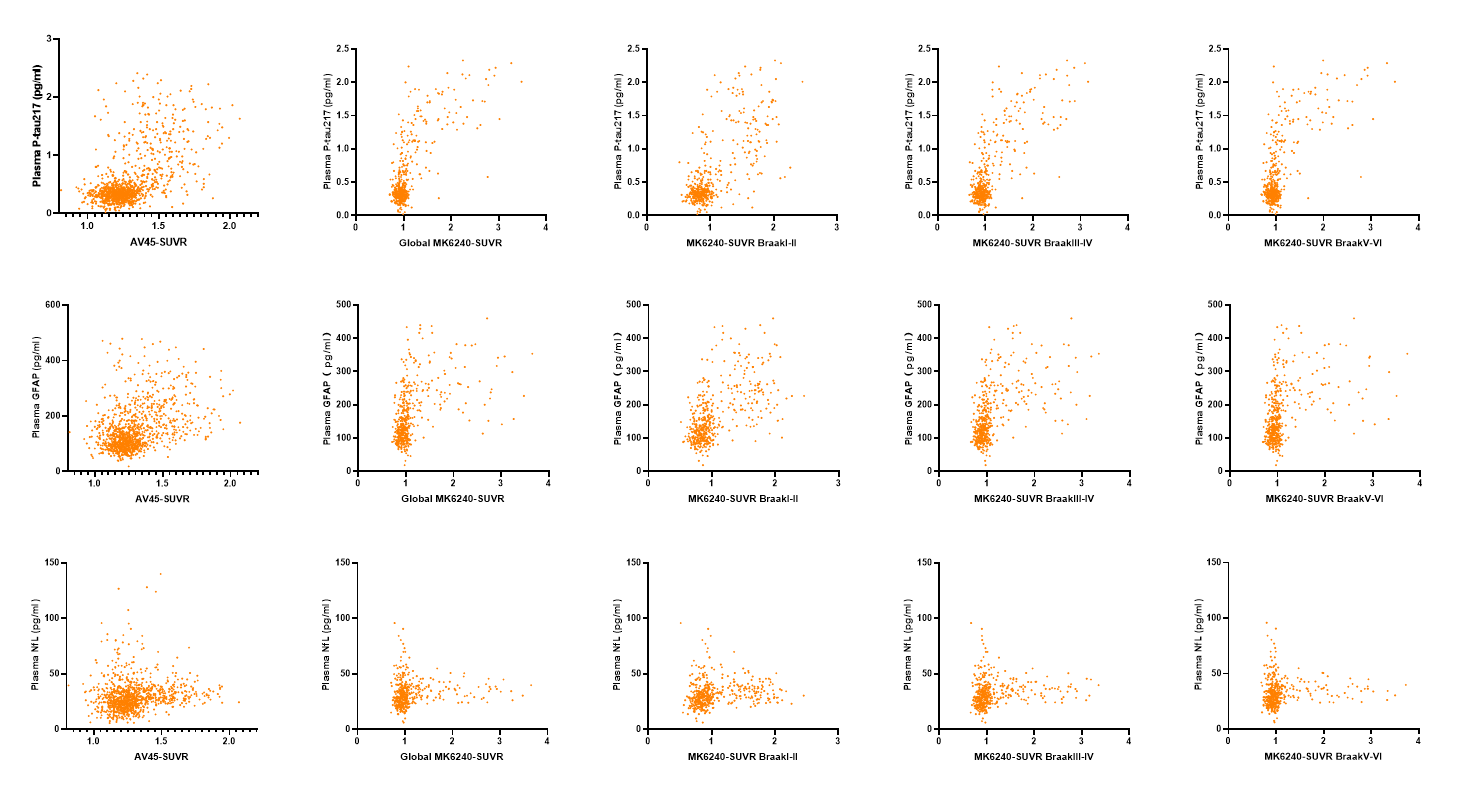


eFigure 2. Scatterplot of plasma biomarkers and AV45-SUVR, as well as MK6240-SUVR in global cortex and specific Braak regions.

**eFigure 2.** Scatterplot of plasma biomarkers and AV45 and MK6240-SUVR in global cortex and specific Braak regions.

| eTable 1. ROC analyses for the plasma biomarkers in discriminating various AT statuses. | | | | | | | | | |
| --- | --- | --- | --- | --- | --- | --- | --- | --- | --- |
| Index | AUC | 95% CI | Cutpoint | Sensitivity  (%) | Specificity  (%) | TP | TN | FP | FN |
| ***Detect A-T-&A-T+ in all participants*** | | | | | | | | | |
| Plasma P-tau217 | 0.931 | 0.907 to 0.950 | ≤0.59 | 95.43 | 83.80 | 376 | 150 | 29 | 18 |
| Plasma GFAP | 0.882 | 0.853 to 0.907 | ≤160.45 | 83.50 | 79.57 | 329 | 148 | 38 | 65 |
| Plasma NfL | 0.708 | 0.667 to 0.745 | ≤26.41 | 45.21 | 87.71 | 165 | 157 | 22 | 200 |
| ***Detect A+T+_Braak III-VI_ in all participants*** | | | | | | | | | |
| Plasma P-tau217 | 0.959 | 0.940 to 0.974 | >0.62 | 93.60 | 90.40 | 117 | 405 | 43 | 8 |
| Plasma GFAP | 0.908 | 0.882 to 0.931 | >172.86 | 84.85 | 82.37 | 112 | 369 | 79 | 20 |
| Plasma NfL | 0.700 | 0.660 to 0.739 | >31.97 | 65.89 | 67.71 | 85 | 281 | 134 | 44 |
| ***Discriminate A+T-&A+T+_BraakI-II_ and A+T+ _BraakIII-VI_ in A+ participants*** | | | | | | | | | |
| Plasma P-tau217 | 0.876 | 0.819 to 0.920 | >0.75 | 88.00 | 74.07 | 110 | 40 | 14 | 15 |
| Plasma GFAP | 0.755 | 0.687 to 0.815 | >205.68 | 71.97 | 68.52 | 95 | 37 | 17 | 37 |
| Plasma NfL | 0.551 | 0.475 to 0.625 | >32.39 | 64.34 | 56.00 | 83 | 28 | 22 | 46 |
| AT statuses are defined by AV45-PET and MK6240-PET on the basis of visual reading. Cutpoints were determined according to the Youden index of the ROC curves. AUC, area under the curve; CL, confident interval;TP, true positive; TN, true negative; FP, false positive; FN, false negative. | | | | | | | | | |

| eTable 2. Effects of plasma P-tau217 and NfL on predicting hippocampal volume, global cognitive decline, and performances across various cognitive domains. | | | | | | | | | | | | | | | | |
| --- | --- | --- | --- | --- | --- | --- | --- | --- | --- | --- | --- | --- | --- | --- | --- | --- |
| Predictors | HV-L | | HV-R | | MoCA-B | | Memory | | Language | | Attention | | Visuospatial | | Execution | |
|  | Beta (95%CI) | VIF | Beta  (95% CI) | VIF | Beta (95% CI) | VIF | Beta (95% CI) | VIF | Beta (95% CI) | VIF | Beta (95% CI) | VIF | Beta (95% CI) | VIF | Beta (95% CI) | VIF |
| ***All participants*** | | | | | | | | | | | | | | | | |
| Plasma P-tau217 | -212.565 (-243.221, -181.909) | 1.150 | -219.817 (-253.455, -186.179) | 1.150 | -2.419 (-2.715, -2.122) | 1.139 | -0.428 (-0.49, -0.366) | 1.161 | -0.194(-0.258, -0.131) | 1.156 | -0.125 (-0.187, -0.063) | 1.163 | -0.098 (-0.165, -0.032) | 1.166 | -0.129 (-0.197, -0.062) | 1.148 |
| Plasma NfL | -35.864 (-68.717, -3.011) | 1.292 | -29.35 (-65.399, 6.699) | 1.292 | -0.984 (-1.294, -0.673) | 1.297 | -0.051 (-0.112, 0.009) | 1.348 | -0.114 (-0.178, -0.051) | 1.355 | -0.053 (-0.113, 0.007) | 1.355 | -0.099 (-0.161, -0.036) | 1.361 | -0.243 (-0.310, -0.177) | 1.352 |
| ***Participants with Aβ-*** | | | | | | | | | | | | | | | | |
| Plasma P-tau217 | -85.458 (-176.806, 5.89) | 1.128 | -52.92 (-152.627, 46.788) | 1.128 | 0.084 (-0.97. 1.138) | 1.147 | -0.093 (-0.268, 0.081) | 1.108 | 0.085 (-0.112, 0.282) | 1.113 | -0.022 (-0.202, 0.157) | 1.111 | 0.190 (0.009, 0.372) | 1.115 | -0.032 (-0.202, 0.139) | 1.109 |
| Plasma NfL | -50.346 (-85.914, -14.777) | 1.234 | -54.539 (-93.363, -15.716) | 1.234 | -1.255 (-1.641, -0.869) | 1.294 | -0.066 (-0.139, 0.006) | 1.293 | -0.103 (-0.183, -0.022) | 1.297 | -0.017 (-0.091, 0.058) | 1.300 | -0.079 (-0.157, 0.000) | 1.302 | -0.168 (-0.239, -0.097) | 1.303 |
| ***Participants with Aβ+*** | | | | | | | | | | | | | | | | |
| Plasma P-tau217 | -181.57 (-239.133, -124.007) | 1.140 | -196.44 (-260.238, -132.642) | 1.140 | -2.242 (-2.702, -1.781) | 1.066 | -0.356 (-0.45, -0.261) | 1.135 | -0.203 (-0.300, -0.106) | 1.093 | -0.117 (-0.206, -0.029) | 1.137 | -0.110 (-0.217, -0.003) | 1.110 | -0.045 (-0.180, 0.090) | 1.122 |
| Plasma NfL | -15.871 (-90.127, 58.386) | 1.258 | 8.697 (-73.603, 90.998) | 1.258 | -0.797 (-1.325, -0.269) | 1.133 | -0.049 (-0.158, 0.06) | 1.231 | -0.145 (-0.251, -0.039) | 1.194 | -0.128 (-0.229, -0.027) | 1.228 | -0.146 (-0.255, -0.036) | 1.207 | -0.414 (-0.572, -0.256) | 1.219 |
| Beta coefficients (95% CI) are for overall tests in linear regressions adjusted for age, sex, and years of education. Plasma P-tau217 and NfL are z-scored. HV, hippocampus volume; VIF, variance inflation factor. | | | | | | | | | | | | | | | | |
